# Case Report: Disseminated Nocardiosis Caused by *Nocardia vulneris* in a Patient With Macroglobulinemia

**DOI:** 10.3389/fpubh.2022.866420

**Published:** 2022-05-10

**Authors:** Fulan Qiu, Zhiyi Ma, Rongrong Zhong, Haonan Huang, Yuehua Wang, Hui Liu

**Affiliations:** ^1^Department of Clinical Laboratory, Fujian Longyan First Hospital, Longyan First Affiliated Hospital of Fujian Medical University, Longyan, China; ^2^Department of Respiration, Fujian Longyan First Hospital, Longyan First Affiliated Hospital of Fujian Medical University, Longyan, China; ^3^Department of Emergency, Fujian Longyan First Hospital, Longyan First Affiliated Hospital of Fujian Medical University, Longyan, China

**Keywords:** disseminated nocardiosis, *Nocardia vulneris*, macroglobulinemia, mass spectrometry, 16S rRNA, linezolid, trimethoprim-sulfamethoxazole, minocycline

## Abstract

This report describes a case of disseminated nocardiosis, caused by *Nocardia vulneris*, in a 61-year-old man with macroglobulinemia and presenting with repeated fever, cough, shortness of breath, and muscle pain. The isolated *Nocardia* strain was resistant to ciprofloxacin, but susceptible to amikacin, gentamicin, tobramycin, linezolid, trimethoprim-sulfamethoxazole, amoxicillin/clavulanic, moxifloxacin, ceftriaxone, cefotaxim, and imipenem. The patient was started on combined meropenem and doxycycline treatment, followed by trimethoprim-sulfamethoxazole, which was subsequently switched to a combination treatment of linezolid, amikacin, and trimethoprim-sulfamethoxazole. The patient recovered, and his condition remained stable. Although infection by *Nocardia vulneris* is rare, and it is easy to miss detection in clinical practice, clinicians should be aware of the possibility of this infection. In addition, the MIC value of the drug sensitivity test should be ascertained when there is a wide choice of medicines. The current case was treated successfully with linezolid, amikacin, and trimethoprim-sulfamethoxazole. In cases of disseminated nocardiosis, the patient should be treated with antimicrobial therapy for at least 12 months. Furthermore, bacteriological examination and antimicrobial susceptibility testing should be performed regularly.

## Introduction

*Nocardia* species are soil saprophytes which are widespread in soil or water, and always associated with pulmonary infection. They can cause serious human infections, especially in immunocompromised patients (1, 2). With the wide use of steroids, immunosuppressants, broad-spectrum antibiotics, and the development of organ transplantation treatment, nocardiosis has been increasingly reported in recent years worldwide (3, 4). About 1 to 179 new cases of Nocardia infections are reported in China each year since 2009, and *Nocardia farcinica* was the most commonly isolated species (39.9%) (4). However, disseminated infections caused by *Nocardia vulneris* are rare and could present with different clinical symptoms. In this study, we report a case of disseminated nocardiosis in a macroglobulinemia patient, with the involvement of lung, brain, blood, and skin, caused by the rarely isolated species *Nocardia vulneris*, which was identified by 16S rRNA sequencing analysis.

## Case Description

**Figure 1:**
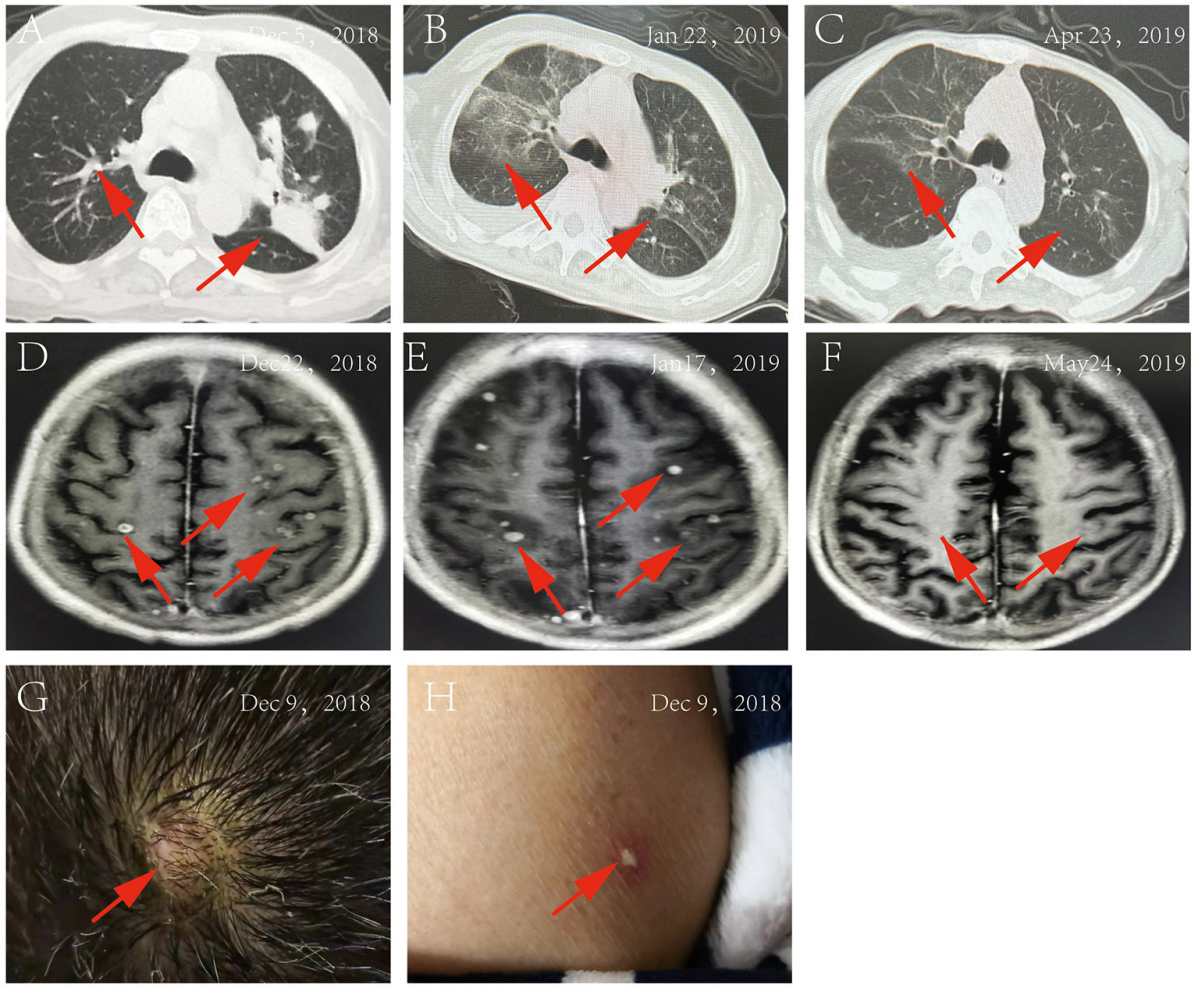
**(A)** Pulmonary CT on December 5, 2018; **(B)** Pulmonary CT on January 22, 2019; **(C)** Pulmonary CT on April 23, 2019; **(D)** MRI of the brain on December 22, 2018; **(E)** MRI of the brain on January 17, 2019; **(F)** MRI of the brain on May 24, 2019; **(G)** the rash on the patient's head; **(H)** The rash on the patient's body.

On 5 December 2018, a 61-year-old man with a 4-year history of macroglobulinemia, undergoing long-term use of prednisone acetate (15 mg/day) and thalidomide (75 mg/night) was sent to our hospital. At the time of presentation, the patient reported a 1-month history of recurrent cough associated with expectoration, repeated fever, and shortness of breath. Within the last week he developed joint pain, muscle pain in the limbs, and a headache. At presentation, a physical examination confirmed that the patient was conscious, and systemic superficial lymph nodes were not affected. Both lungs sounded clear, with no dry or wet rale. Rhythm of the heart was regular, with no murmurs. The abdomen was soft, with no tenderness and rebound tenderness; spine tenderness/pain was observed, without percussion pain; limb muscle tenderness without rebound tenderness was reported and each joint presented as normal. No obvious pitting edema was observed in either of the lower limbs, pathological character was negative. The patient was admitted right in the middle of the fever break, and his admission examination showed a body temperature of 36.3°C, respiration at 20 times/min, pulse 92 beats/min, pulmonary CT: multiple nodular shadows or lamellar shadows in the left lung, new appearance of multiple lymph nodes in bilateral axilla and mediastinum, and partial lymph node swelling, as shown in Figure 1A. Laboratory findings were as follows: blood routine examination: WBC, 3.35 × 10^9^/L; neutrophils, 58.8%; hemoglobin, 115 g/L; blood platelet, 193 × 10^9^/L; urine routine: urine protein, 1+; urine sugar, 1+; urine protein, 633.5 mg/L; immunoglobulin IgA, 0.51 g/L; immunoglobulin IgG, 4.54 g/L; immunoglobulin IgM, 6.15 g/L; D-dimer, 1.16 mg/L; C-reactive protein (CRP), 192.78 mg/L; total protein, 47.5 g/L; albumin, 27.5 g/L; and procalcitonin (PCT), 0.54 ng/ml, as shown in Table 1. Levofloxacin was administered for 3 days. On December 7, the patient developed fever with a peak value of 39.5°C. On December 8, the patient retained high fever, with a WBC value of 1.09 × 10^9^/L, and the absolute value of neutrophils was 0.41 × 10^9^/L. Thereafter, the patient discontinued treatment with steroids and immunosuppressants, and treated with recombinant human granulocyte stimulating factor (90 ug/day) to increase white blood cells. In addition, meropenem (1 g/day), combined with doxycycline (100 mg/day), was administered. On December 9, two peanut-sized nodules developed, with a moderate hardness in the scalp. The right temporal area was accompanied by an ulcer and obvious tenderness. Multiple reddish plaque and nodules were scattered over the trunk and limbs, with tenderness, and some erythematous pustules were observed, as shown in Figures 1G,H. Gram-positive bacilli (suspected *Nocardia*) were found in the blood culture, and further 16S rRNA gene identification confirmed the isolate as *Nocardia vulneris*. Based on the results of drug sensitivity testing, doxycycline was replaced with trimethoprim-sulfamethoxazole (3 g/day). Subsequently, the sputum smear, bronchial washing fluid and scalp all revealed the presence of *Nocardia*. MRI revealed the presence of a brain abscess and multiple nodular shadows as shown in Figure 1D, and the patient's fever peaked at 40°C.

**Table 1:** Clinical information of the patient during hospitalization.

**Date**	**Dec 5, 2018**	**Jan 22, 2019**	**April 23, 2019**	**Reference range**
Day after onset	30	48	90	
WBC	3.35 × 10^9^/L	3.84 × 10^9^/L	2.83 × 10^9^/L	3.5 × 10^9^/L 9.5 × 10^9^/L
Neutrophils	58.8%	67.2%	63.2%	40 70%
Hemoglobin	115 g/L	79 g/L	75 g/L	130 175 g/L
Blood platelet	193 × 10^9^/L	93 × 10^9^/L	126 × 10^9^/L	125 × 10^9^/L 350 × 10^9^/L
Urine protein	1+	1+	+-	negative
Urine sugar	1+	negative	negative	negative
immunoglobulin IgA	0.51 g/L	/	0.32 g/L	0.7 4.0 g/L
immunoglobulin IgG	4.54 g/L	/	8.37 g/L	7.0 16.0 g/L
immunoglobulin IgM	6.15 g/L	/	10.8 g/L	0.4 2.3 g/L
D-dimer	1.16 mg/L	1.02 mg/L	1.17 mg/L	0 1 mg/L
C-reactive protein	192.78 mg/L	49.43 mg/L	33.21 mg/L	0.06 8.2 mg/L
Total protein	47.5 g/L	56.0 g/L	55.0 g/L	65 85 g/L
Albumin	27.5 g/L	28.2 g/L	31.3 g/L	40 55 g/L
Procalcitonin	0.54 ng/ml	0.32 ng/ml	1.3 ng/ml	<0.5 ng/ml

On 12 December, trimethoprim-sulfamethoxazole (3 g/day) treatment, in combination with ceftriaxone (2 g/day), was started. However, the patient's symptoms did not abate. On 14 December, treatment was changed to SMZco combined with linezolid. One day later, the patient's temperature remained unchanged, and according to the drug susceptibility analysis, the MIC values of linezolid, amikacin, and trimethoprim-sulfamethoxazole were confirmed and the treatment was changed to the combination of linezolid, amikacin, and trimethoprim-sulfamethoxazole. On 16 December, there was a noticeable drop in the temperature of the patient, and the PCT and CRP significantly decreased. On 24 December, the patient displayed normal body temperature, with no obvious chills, occasional cough and shortness of breath, with production of phlegm. Skin rash and scalp abscess became narrowed, both lungs sounded clear with a little wet rale. Antibiotics were changed to trimethoprim-sulfamethoxazole combined with linezolid, after which the symptoms gradually improved. Multiple microbial cultures of the blood, phlegm, and pus were negative and the lung CT showed absorption of the lesions Figure 1B, MRI showed an increased presence of the multiple nodular shadows as shown in Figure 1E. On January 28, the patient was discharged from the hospital, with the instruction to continue long-term oral trimethoprim-sulfamethoxazole combined minocycline for 12 months. Follow-up lung CT Figure 1C and brain MRI Figure 1F showed that the lesions had improved significantly.

## Diagnostic Assessment

### Bacterial Culture and Identification

**Figure 2:**
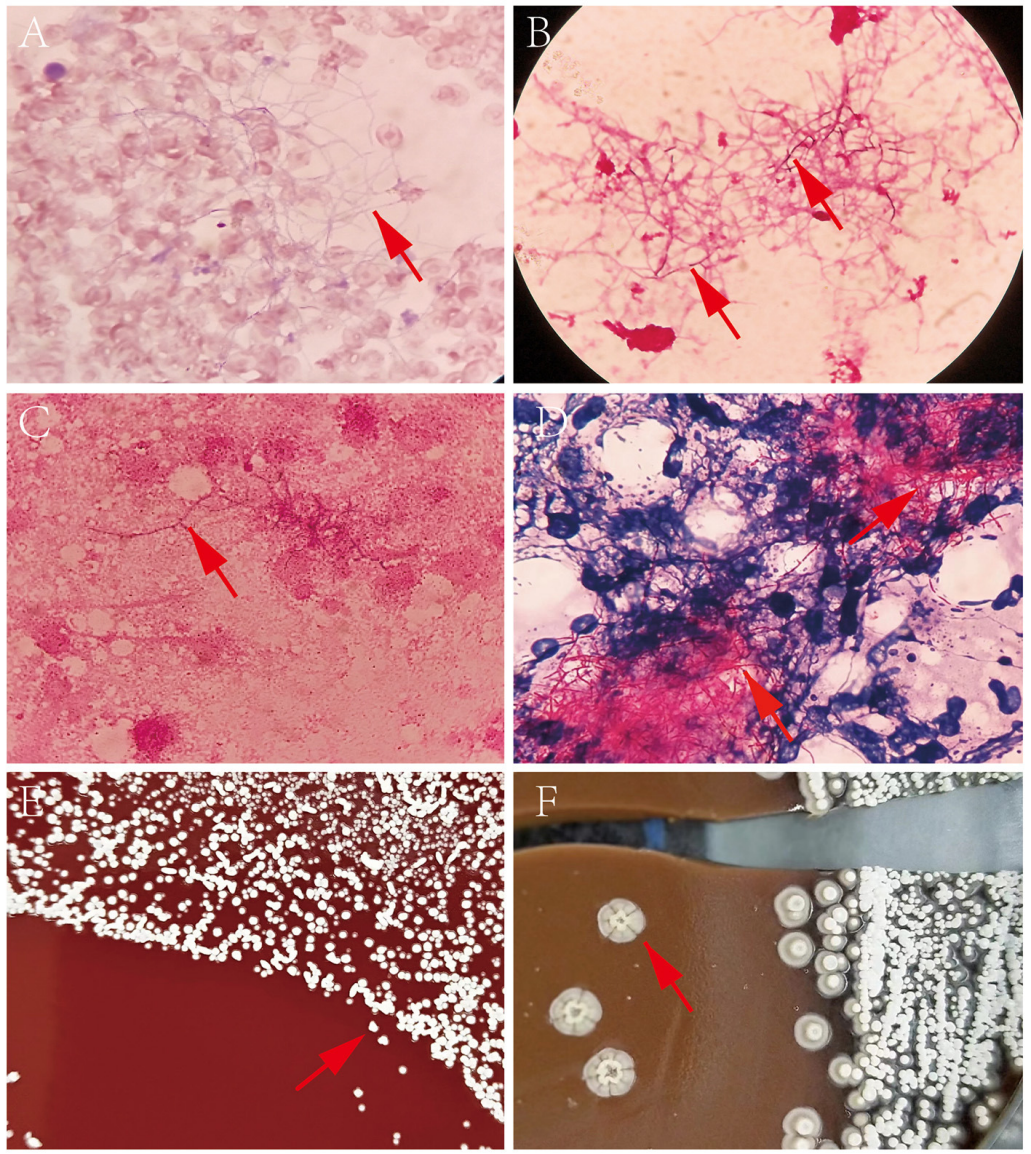
**(A)** Direct blood culture smear by Wright-giemsa staining (×1,000); **(B)** Direct blood culture smear by Gram staining (×1,000); **(C)** Nocardia vulneris by Gram staining; **(D)** Nocardia vulneris by weak acid fast staining; **(E)** Colonies of Nocardia vulneris cultured for 3 days; **(F)** Colonies of Nocardia vulneris cultured for 15 days.

A set of blood culture was sent for detection on 7 December, and the blood culture was positive after 35 h. Gram-positive long thin filamentous bacterial group was found in the smear, as shown in Figures 2A,B. Pin-sized white colonies could be seen on the transferred blood plate after 24 h. Large, dry, white colonies with obvious smell of dust, which did not embed into the agar, was observed after 72 h Figures 2E,F. Sputum and pus samples were inoculated into blood plate and cultured at 35°C for 48 h. No growth of *Nocardia* was observed; however, fine colonies were observed after culture for 72 h. Meanwhile, long filamentous Gram-positive bacterial group was found in sputum samples and scalp pus, and the weak acid-fast staining was positive, as shown in Figures 2C,D. The colony was identified as *Nocardia brasiliensis* by mass spectrometry system Bruker (Bruker Company, Bruker MALDI Biotyper TOF, America) and VITEK MS (BioMerieux Company, BioMerieux VITEK MS, China), with scores of 1.718, and 2.0, respectively. However, this isolate was identified as *Nocardia vulneris* by 16S rRNA gene sequencing analysis (GenBank NR148265.1). The universal primers used for sequencing were (27F/1492R; 5′-AGAGTTTGATCCTGGCTCAG-3′/5′-CTACGGCTACCTTGTTACGA-3′), with the cycling conditions of initial denaturation 94°C for 5 min, 25 cycles of denaturation 94°C for 30 sec, annealing 57°C for 30 sec, extension 72°C for 90 sec, and a final extension 72°C for 5 min (RuiBo Biotechnology Company, Beijing, China); or used the primer (7F/1540R; 5′-CAGAGTTTGATCCTGGCT-3′/5′-AGGAGGTGATCCAGCCGCA-3′), with the cycling conditions of initial denaturation 94°C for 4 min, 30 cycles of denaturation 94°C for 45 sec, annealing 55°C for 45 sec, extension 72°C for 1 min, and a final extension 72°C for 10 min (Sangon Biotechnology Company, Shanghai, China).

### Drug Sensitivity Test

The trace broth dilution method was used to determine the sensitivity of the strain to antibiotics, and CLSI M24 was used as the standard for determining drug sensitivity results, as shown in Table 2.

**Table 2:** Susceptibility of *Nocardia vulneris* isolate to different antimicrobials.

**Antimicrobials**	**MIC**	**Susceptibility**
Trimethoprim-sulfamethoxazole	≤0.5/9.5	S
Amoxycillin/clavulanic acid	≤2/1	S
Amikacin	2	S
Gentamicin	≤1	S
Tobramycin	≤1	S
Imipenem	4	S
Linezolid	1	S
Ciprofloxacin	≥8	R
Moxifloxacin	1	S
Ceftriaxone	8	S
Cefotaxim	8	S
Cefepime	16	I
Minocycline	2	I

*MIC, minimum inhibitory concentration; S, susceptible; I, intermediate; R, resistant. Susceptibility of the isolate to antimicrobials was defined according to the CLSI M24-A guidelines*.

## Discussion

As a soil saprophyte, *Nocardia* species invades the human body through the respiratory tract, skin, or digestive tract, resulting in the development of nocardiosis. Species of *Nocardia* involved in human pathogenesis include *Nocardia brasiliensis, Guinea pig nocardia* and *Picrax nocardia*. Nocardiosis is a life-threatening disease (5) usually found in immunocompromised patients, or populations where it occurs secondary to other diseases. The lung is the most common site of nocardiosis, accounting for about 70–80% (6, 7). Disseminated infections caused by *Nocardia* usually affect immunocompromised patients (8), and typically originate in the lungs, then spread to the brain. However, an individual with normal immune function and disseminated nocardiosis which originated in the lungs and spread to the brain, causing brain abscess, has been reported (9).

Nocardia bacteremia is an extremely severe form of disseminated nocardiosis, the mortality rate may account for approximately 60% (10). However, to the best of our knowledge, *Nocardia vulneris* always manifests as skin and/or subcutaneous tissue infections, and though there are no previous reports on its role in disseminated infections, here, we present a case of disseminated *Nocardia vulneris* infection.

*Nocardia* do not constitute as a part of the human normal flora; therefore, it can be diagnosed as nocardiosis when these organisms are isolated from sputum, alveolar lavage fluid, blood, pus, pleural fluid, subcutaneous tissues, or other samples. In the case reported here, the patient was undergoing immunosuppressant and steroid treatment for macroglobulinemia for 4 years. He was admitted to the hospital suffering from a 1-month history of repeated cough associated with sputum, repeated fever, and shortness of breath. In addition, the patient reported a 1-week history of limb muscle pain, interphalangeal joint pain, and headache. A blood culture was started upon admission, and the aerobic blood culture was positive after 35 h. Gram-positive red filamentous branching bacterial groups were found in the smear. Although visible in the phlegm or alveolar lavage fluid direct smear specimens, stained by wright-giemsa staining and weak acid-fast dye, it is also easily be concealed by other bacteria which grow rapidly, so microscopic examination of the smear is very important in early diagnosis. In addition, *Nocardia* grow slowly, usually taking 72 h to form small colonies, so medical personnel should extend the time of bacteria culture to avoid the problems of misdiagnosis. Of note, small microflora could be seen which came from the positive blood culture, and were transferred to the blood plate for 24 h. This may be the reason that nutrient solution in the blood cultures could neutralize the antibiotics or antibacterial factors which remained in the specimen and made bacteria grow quickly. Because it is time-consuming and difficult for the traditional microbial identification method to identify the species of *Nocardia*, it does not meet the needs of the accurate and rapid clinical identification of pathogenic bacteria. However, mass spectrometry, and other modern molecular biology technologies, especially PCR and gene sequencing, could provide a good foundation for rapid identification of *Nocardia* species. A recent report has shown that 91% of the *Nocardia* species could be accurately identified by the mass spectrometry system VITEK MS (11). In this case, the species identified by mass spectrometry and 16S rRNA gene sequencing was incorrect, possibly because the species *Nocardia vulneris* was not in the library of the mass spectrometry system Bruker (Bruker Company, Bruker MALDI Biotyper TOF, America) and VITEK MS (BioMerieux Company, BioMerieux VITEK MS, China), so the organism was identified as *Nocardia brasiliensis* by Bruker and VITEK MS. This report suggests that 16S rRNA gene sequencing method should be adopted, and the library of the mass spectrometry system should be upgraded frequently or combined with the results of gene sequencing.

Sulfonamides have long been considered the first line of drug treatment in nocardiosis; however, a report regarding the antibiotic susceptibility of *Nocardia* showed that the resistance rate of *Nocardia* against sulfonamides is 57%, and the mortality rate is higher when it is used alone (12). For immunosuppressed patients or patients with disseminated infections, trimethoprim-sulfamethoxazole monotherapy has been changed to amikacin combined with imipenem or linezolid, which is for central nervous system infections or multidrug-resistant bacterial infections (13–15). In this case, as the results of the direct blood culture smear were received that pointed to suspected *Nocardia* infection, the patient was treated by trimethoprim-sulfamethoxazole combined with meropenem for 3 days. Given that the patient still displayed a high fever, the treatment was changed to trimethoprim-sulfamethoxazole combined with ceftriaxone for 2 days, but the patient's symptoms persisted. Following treatment with trimethoprim-sulfamethoxazole combined with linezolid for 1 day, which showed no efficacy, the MIC values of linezolid, amikacin, and trimethoprim-sulfamethoxazole were confirmed and the treatment was changed to the combination of linezolid, amikacin and trimethoprim-sulfamethoxazole due to the severity of the illness. Concomitantly, because the patient had extremely poor renal function, the clinical pharmacists reduced the dosage of amikacin to prevent further renal function damage; the patient's temperature showed a decreasing trend after treatment with trimethoprim-sulfamethoxazole combined with linezolid and amikacin for 1 day. After treatment for 15 days, the patient's infection symptoms had largely resolved. Thereafter, the patient was treated with an oral combination of trimethoprim-sulfamethoxazole and minocycline for 6 months, without any relapse after treatment cessation. The successful treatment of this case fully demonstrates that the MIC value of the drug sensitivity test should be ascertained when there is a wide choice of drugs available. In addition, multidisciplinary cooperation may be warranted and can effectively significantly improve the success rate of treatment. Scott *et al*. reported a case in which retinitis caused by *Nocardia veterana* was successfully treated by a combination of linezolid, amikacin, and meropenem (16). For the cases which are diagnosed, patients should be treated for at least 12 months with antimicrobial therapy. Bacteriological examination and antimicrobial susceptibility testing should be performed regularly. In addition, according to the antimicrobial sensitivity testing, the treatment regimen should be adjusted with the appropriate monitoring, and follow-ups are required to determine whether the patient relapsed after cessation of the treatment.

## Conclusion

In conclusion, this is the first reported case of disseminated *Nocardia vulneris* infection in a macroglobulinemia patient. Despite the clinical characteristics, diagnostic methods and optimal treatment protocols for *Nocardia vulneris* remain unclear because of its rarity. Our findings provide the information that 16S rRNA gene sequencing method should be adopted, or gene sequencing combined with the results of mass spectrometry, to diagnose *Nocardia vulneris* infection. In addition, we suggest that the MIC value of the drug sensitivity test should be ascertained when there is a wide choice of drugs available, and disseminated cases should be treated with at least 12 months of antimicrobial therapy, and regular bacteriological examination and antimicrobial susceptibility testing.

## Data Availability Statement

The original contributions presented in the study are included in the article/supplementary materials, further inquiries can be directed to the corresponding author.

## Ethics Statement

Written informed consent was obtained from the individual(s) for the publication of any potentially identifiable images or data included in this article.

## Author Contributions

FQ: data analysis, data interpretation, and writing. ZM: study design, data collection, and data interpretation. RZ: data analysis and data interpretation. H-nH: figures and data collection. YW: pictures. HL: literature search, writing, and funds collection. All authors contributed to the article and approved the submitted version.

## Funding

This work was sponsored by Longyan City Science and Technology Plan Project (Grant No. 2021LYF17034).

## Conflict of Interest

The authors declare that the research was conducted in the absence of any commercial or financial relationships that could be construed as a potential conflict of interest.

## Publisher's Note

All claims expressed in this article are solely those of the authors and do not necessarily represent those of their affiliated organizations, or those of the publisher, the editors and the reviewers. Any product that may be evaluated in this article, or claim that may be made by its manufacturer, is not guaranteed or endorsed by the publisher.
